# Unusual clinical phenotype of Stargardt disease

**DOI:** 10.5935/0004-2749.20210064

**Published:** 2021

**Authors:** Pedro Molina-Solana, María José Morillo-Sánchez, Cristina Méndez-Vidal, Manuel Ramos-Jiménez, Borja Domínguez-Serrano, Guillermo Antiñolo, Enrique Rodríguez-de-la-Rúa-Franch

**Affiliations:** 1 Department of Ophthalmology, University Hospital Virgen Macarena, Seville, Spain; 2 Department of Maternofetal Medicine, Genetics and Reproduction, Institute of Biomedicine of Seville, University Hospital Virgen del Rocío/CSIC/University of Seville, Seville, Spain; 3 Rare Diseases Networking Biomedical Research Centre (CIBERER), Seville, Spain; 4 Department of Clinical Neurophysiology, University Hospital Virgen Macarena, Seville Spain; 5 Retics Oftared, Institute Carlos III, Madrid, Spain

**Keywords:** Stargardt disease/diagnosis, Retinal dystrophies, ATP-binding cassette transporter, subfamily A, member 4, Tomography, optical coherence, Electroretinography, Fluorescein angiography, Doença de Stargardt/diagnóstico, Distrofias retinianas, Membro 4 da Subfamília A de transportadores de cassetes de ligação de ATP, Tomografia de coerência óptica, Eletrorretinografia, Angiofluoresceinografia

## Abstract

Mutations in the *ABCA4* gene are a common cause of Stargardt disease; however, other retinal phenotypes have also been associated with mutations in this gene. We describe an observational case report of an unusual clinical phenotype of Stargardt disease. The ophthalmological examination included best corrected visual acuity, color and autofluorescence photography, fluorescein angiography, optical coherence tomography, and electrophysiology tests. Targeted next-generation sequencing of 99 genes associated with inherited retinal dystrophies was performed in the index patient. A 48-year-old woman presented with a best corrected visual acuity of 20/25 and 20/20. Fundoscopy revealed perifoveal yellow flecked-like lesions. Fluorescein angiography and fundus autofluorescence findings were consistent with pattern dystrophy. Pattern electroretinogram demonstrated bilateral decrease of p50 values. Genetic testing identified two heterozygous missense mutations, c.428C>T, p.(Pro143Leu) and c.3113C>T, p.(Ala.1038Val), in the *ABCA4* gene. Based on our results, we believe that these particular mutations in the *ABCA4* gene could be associated with a specific disease phenotype characterized by funduscopic appearance similar to pattern dystrophy. A detailed characterization of the retinal phenotype in patients carrying specific mutations in *ABCA4* is crucial to understand disease expression and ensure optimal clinical care for patients with inherited retinal dystrophies.

## Abstract (pt)

Mutações no gene *ABCA4* são causa comum da doença de Stargardt, mas outros fenótipos da retina também foram associados a mutações nesse gene. Apresentamos um relato de caso observacional de um fenótipo clínico incomum da doença de Stargardt. O exame oftalmológico incluiu a acuidade visual com melhor correção, fotografia em cores e com autofluorescência, angiofluoresceinografia, tomografia de coerência óptica e testes de eletrofisiologia. Na paciente em questão, realizou-se o sequenciamento de próxima geração de 99 genes associados a distrofias retinais hereditárias. Tratava-se de uma mulher de 48 anos com melhor acuidade visual corrigida de 20/25 e 20/20. A fundoscopia revelou lesões puntiformes amarelas perifoveais. Os resultados da angiofluoresceinografia e da autofluorescência do fundo de olho foram consistentes com distrofia em padrão. A eletrorretinografia por padrões mostrou diminuição bilateral dos valores de p50. Os testes genéticos revelaram duas mutações *missense* heterozigóticas, c.428C>T, p. (Pro143Leu) e c.3113C>T, p. (Ala.1038Val), no gene *ABCA4*. Nossos resultados nos fazem pensar que essas mutações específicas em *ABCA4* talvez possam estar associadas a um fenótipo específico da doença, caracterizado por uma aparência fundoscópica semelhante à da distrofia em padrão. Uma caracterização detalhada do fenótipo da retina em pacientes portadores de mutações específicas em *ABCA4* é crucial para compreender a expressão da doença e para garantir o tratamento clínico ideal para pacientes com distrofias retinais hereditárias.

## INTRODUCTION

Autosomal recessive Stargardt disease (STGD1; MIM 248200) is the most common inherited macular dystrophy in both children and adults, which is caused by pathogenic variants in the ATP-binding cassette transporter type A4 (*ABCA4*) gene^(1)^. Individuals affected with STGD1 exhibit variable age at onset and heterogeneous phenotypes, with the early-onset group (generally observed before the age of 10 years) experiencing the most severe phenotype, clinically resembling severe autosomal recessive cone-rod dystrophy (arCRD)^(1-3)^. Different combinations of *ABCA4* variants have been suggested to explain the different phenotypes, including other macular dystrophies such as pattern dystrophy^(3-5)^, and the degree of severity of *ABCA4*-associated retinopathies. The combination of frequent, low-penetrant variants and severe variants, or two moderately severe variants, has been associated with a milder, late-onset disease, whereas a combination of moderately severe and severe variants or two severe variants has been proposed to cause early-onset Stargardt disease or arCRD^(6-7)^.

## CASE REPORT

**Figure 1:**
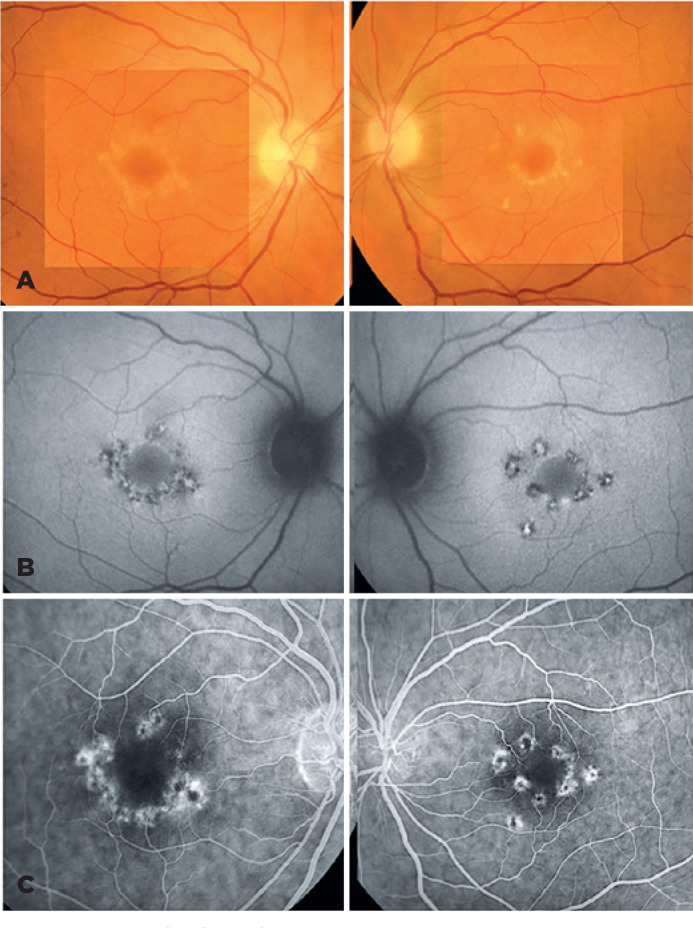
Ophthalmological examination A) Retinography showed perifoveal yellowish fleck-like lesions encircling the foveal area. B) AF revealed intense hyperautofluorescent rounded lesions (corresponding to the yellowish flecks) compatible with accumulation of liposfuscin-like material surrounded by an hypoautofluorescent halo corresponding to electroretinography (RPE) atrophy C) FA disclosed perifoveal rounded hypofluorescent lesions surrounded by hyperfluorescent areas both isolates and confluent (RPE atrophy) simulating argon photocoagulation laser impacts.

**Figure 2:**
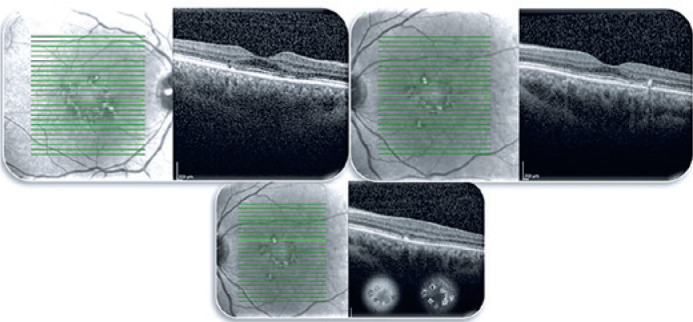
SD-OCT image showed material in the subretinal space between the photoreceptors and RPE. They appear as melanolipofuscin accumulation in AF and hypofluorescence in FA.

We report the case of a 48-year-old woman who presented for a routine ophthalmoscopic examination. All investigations were performed according to the tenets of the Declaration of Helsinki with approval from the Institutional Review Board of the University of Tuebingen. The routine ophthalmoscopic examination included best corrected visual acuity (BCVA, Snellen 20 feet), which for our patient was 20/25 in the right eye and 20/20 in the left eye. Color and autofluorescence fundus (AF) photographs after pupil dilation using 1% tropicamide and fundus fluorescein angiography (FA) (Topcon Model TRC-50DX, Topcon Medical System, Oakland, NJ, USA) are depicted in figure 1, which highlight how the foveal area is perfectly preserved from the material accumulation in all three images. Moreover, spectral-domain optical coherence tomography (SD-OCT) macular scans images were taken (Heidelberg Engineering, Heidelberg, Germany) as shown in figure 2, which indicates the presence of foveal spare as well. A minimally altered photoreceptor layer is observed in the fovea explaining the low visual loss, which the patient had not realized until the measurement of BCVA. All these findings revealed an entity similar to a pattern dystrophy, which was our first option in the differential diagnosis. Electrophysiology tests were performed according to the recommendations of the International Society for Electrophysiology of Vision (ISCEV), and the results were more probably against the diagnosis of a pattern dystrophy. Electrooculography revealed a ratio of the light peak to dark trough or an Arden ratio >1.8 bilaterally (within normal limits, which could also correspond to a pattern dystrophy in an early stage), full-field electroretinography (ERG) revealed normal scotopic and photopic responses, and pattern ERG disclosed bilateral decrease of p50 values. On the basis of these results, we can conclude the presence of a bilateral macular affectation without diffuse involvement of retinal-dependent responses and with preservation of integrity of the outer retinae (pigmentary epithelium - photoreceptor outer segment).

**Figure 3:**
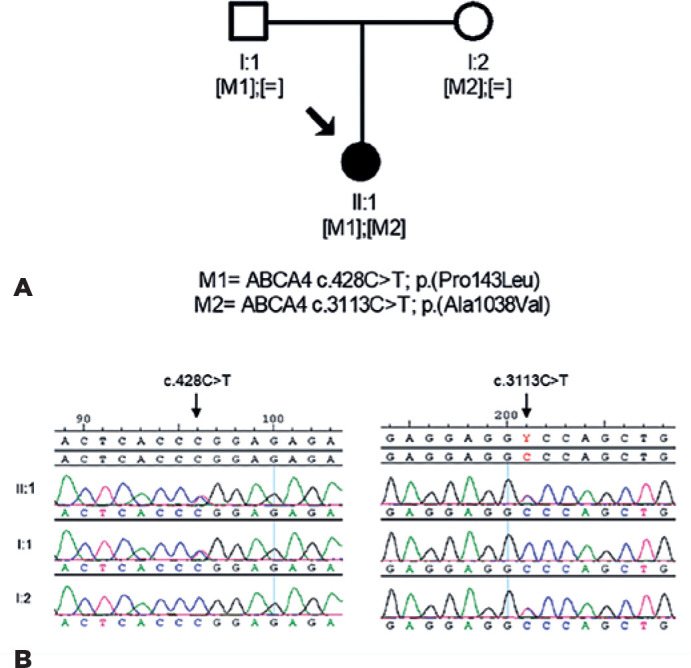
Genetic diagnosis of the analyzed family. A) Pedigree of the family showing the co-segregation analysis results. B) Sanger sequencing confirming the presence of two compound heterozygous *ABCA4* variants, c.3113C>T (exon 4) and c.428C>T (exon 21), in affected individual (II:1) and the heterozygous variant in her parents (I:1 and I:2).

Genetic testing in the index patient using targeted next-generation sequencing (SeqCap^®^ EZ Choice Enrichment kit, Roche NimbleGen and the Illumina NextSeq500 sequencer) of 99 genes associated with inherited retinal dystrophies (IRD) (Table 1) revealed two compound heterozygous variants, c.428C>T, p.(Pro143Leu) and c.3113C>T, p.(Ala.1038Val), in the *ABCA4* gene, typically altered in Stargardt disease. No additional candidate variants were identified in the IRD-related genes examined in this study. Segregation analysis showeed that each of the parents was heterozygous for one of the two variants (Figure 3), which have been previously reported as pathogenic (c.3113C>T) or likely pathogenic (c.428C>T) mutations in public databases (ClinVar Variation ID 7894 and ID 99273, respectively; accessed October 28, 2019).

**Table 1:** List of genes included in the capture inherited retinal dystrophies (IRD) panel

Gene	RefSeq	Gene	RefSeq	Gene	RefSeq
*ABCA4*	NM_000350	*FBN2*	NM_001999	*PRPF31*	NM_015629
*ABHD12*	NM_015600	*FSCN2*	NM_001077182	*PRPF8*	NM_006445
*ADGRV1*	NM_032119	*GUCA1A*	NM_000409	*PRPH2*	NM_000322
*AIPL1*	NM_014336	*GUCA1B*	NM_002098	*RAB28*	NM_001017979
*ALMS1*	NM_015120	*GUCY2D*	NM_000180	*RBP3*	NM_002900
*ARL6*	NM_177976	*HK1*	NM_033497	*RD3*	NM_001164688
*BBS1*	NM_024649	*IMPDH1*	NM_000883	*RDH12*	NM_152443
*BBS10*	NM_024685	*INVS*	NM_014425	*RGR*	NM_002921
*BBS12*	NM_152618	*LCA5*	NM_001122769	*RHO*	NM_000539
*BBS2*	NM_031885	*LRAT*	NM_004744	*RLBP1*	NM_000326
*BEST1*	NM_001139443	*MERTK*	NM_006343	*ROM1*	NM_000327
*C1QTNF5*	NM_015645	*MFRP*	NM_031433	*RP1*	NM_006269
*C2orf71*	NM_001029883	*MFSD8*	NM_152778	*RP1L1*	NM_178857
*CA4*	NM_000717	*MKKS*	NM_170784	*RP2*	NM_006915
*CACNA1F* NM_001256789		*MYO7A*	NM_000260	*RP9*	NM_203288
*CDH23*	NM_022124	*NMNAT1*	NM_022787	*RPE65*	NM_000329
*CDHR1*	NM_033100	*NPHP1*	NM_001128178	*RPGR*	NM_001034853
*CEP250*	NM_007186	*NPHP4*	NM_015102	*RPGRIP1*	NM_020366
*CEP290*	NM_025114	*NR2E3*	NM_016346	*RS1*	NM_000330
*CERKL*	NM_201548	*NRL*	NM_006177	*SAG*	NM_000541
*CFH*	NM_000186	*OAT*	NM_000274	*SAMD11*	NM_152486
*CHM*	NM_000390	*OFD1*	NM_003611	*SNRNP200*	NM_014014
*CIB2*	NM_006383	*PAX6*	NM_001258462	*TIMP3*	NM_000362
*CLRN1*	NM_052995	*PCDH15*	NM_001142763	*TOPORS*	NM_005802
*CNGA1*	NM_001142564	*PDE6A*	NM_000440	*TULP1*	NM_003322
*CNGA3*	NM_001298	*PDE6B*	NM_000283	*UNC119*	NM_005148
*CNGB1*	NM_001297	*PDE6C*	NM_006204	*USH1C*	NM_153676
*CNGB3*	NM_019098	*PDZD7*	NM_001195263	*USH1G*	NM_173477
*COL2A1*	NM_001844	*PNPLA6*	NM_001166111	*USH2A*	NM_206933
*CRB1*	NM_201253	*POMGNT1* NM_001243766		*VCAN*	NM_004385
*CRX*	NM_000554	*PRCD* NM_001077620		*WHRN*	NM_015404
*EYS*	NM_001142800	*PROM1* NM_006017		*ZNF408* NM_001184751	
*FAM161A* NM_001201543		*PRPF3* NM_004698			

## DISCUSSION

Identifying novel genotype-phenotype relationships is currently a major area of interest. In the current report, we suggest a novel correlation between the presence of *ABCA4* variants and the development of an unusual clinical phenotype of Stargardt disease. Although genetic disorders are, in general, individually rare, and obtaining sufficient number of cases is not always possible, additional cases with a similar genotype-phenotype correlation should be recruited and analyzed for establishing reliable genotype-phenotype correlations.

Previous studies have proposed a genotype-phenotype correlation model for *ABCA4* variants in which, depending on the mild or severe nature of these variants and the residual activity of the mutant protein, the clinical phenotypes can range from a mild, late-onset disease to early-onset, more severe disorders^(6-7)^. Although the c.3113C>T variant is significantly enriched in Caucasian patients with retinal dystrophy, it has been considered as a mild allele as it was not detected in a homozygous state in patients, although this was expected based on its high frequency in the Exome Aggregation Consortium database (ExAC; http://exac.broadinstitute.org/). Moreover, the presence of two homozygous individuals in the control population confirmed the mild nature of this variant. In contrast, although reported in ClinVar as a likely pathogenic variant, the pathogenicity of c.428C>T remains controversial. This variant has not been found to be significantly enriched in patients with STGD1, although its frequency is higher in the cohort than in the control population. Furthermore, no homozygous healthy individuals have been described till date, whereas one individual with STGD1 was reported to be homozygous for the c.428C>T variant^(8)^. This finding argues for a relatively severe effect of c.428C>T. These results together with the family segregation studies suggest that this variant is pathogenic and the cause, together with c.3113C>T, of the retinal phenotype in this patient. Considering the mild loss of vision and the age of the patient, which indicates a chronic slow progressive course of retinopathy and in addition to the fundus and OCT appearance, where the accumulation of lipofuscin at the level of the retinal pigment epithelium is a typical characteristic feature^(9)^, pattern dystrophy could be a possible diagnosis^(10)^ or as in this case, like another macular dystrophy phenotypically simulating a pattern dystrophy. Our findings emphasize the clinical complexity of *ABCA4*-associated diseases. Analysis of a larger series of cases at the clinical and genetic levels would certainly help us and be indispensable for understanding this unusual phenotype of Stargardt disease.

This study conformed to the tenets of the Declaration of Helsinki (Edimburgh, 2000) and was approved by the Institutional Review Boards of the Hospitals Virgen del Rocio and Virgen Macarena, Seville. An informed consent form was signed by all participants for clinical and molecular genetic studies (PI15_01648 and CTS1664).

The patient has consented to the submission of the case report to the journal.
